# Desmoplastic fibroblastoma (collagenous fibroma) of the oral cavity

**DOI:** 10.4317/jced.52605

**Published:** 2016-02-01

**Authors:** Thais-dos Santos-Fontes Pereira, Júlio-Cesar-Tanos de Lacerda, Michelle-Danielle Porto-Matias, Alessandro-Oliveira de Jesus, Ricardo-Santiago Gomez, Ricardo-Alves Mesquita

**Affiliations:** 1 Department of Oral Surgery and Pathology. School of Dentistry. Universidade Federal de Minas Gerais; 2 Stomatology service, Odilon Behrens Hospital, Belo Horizonte, Minas Gerais, Brazil

## Abstract

Desmoplastic fibroblastoma is benign soft tissue tumor, with fibroblastic or myofibroblastic origin, that rarely occurs in oral cavity. We reported the case of a 56-year-old man who presented a tumor in the left mandibular alveolar ridge, with slow and asymptomatic growth, with no osseous involvement. The tumor was sessile with lobulated surface, covered by healthy mucosa with erythematous areas. The lesion was excised and specimens sent to histopathology and immunohistochemistry. Histopathological exam showed a non-encapsulated fibroblastic proliferation, characterized by myofibroblasts, spindle and stellate fibroblasts with large or oval nuclei and bi or tri nucleation, immersed in an abundant hypocellular dense collagen stroma. Tumor cells were positive for vimentin, HHF35, α-smooth muscle actin and factor XIIIa. The diagnosis of desmoplastic fibroblastoma was based in the clinical history of absence of trauma related to the growth in the alveolar ridge, associated with macroscopic, microscopic and immunohistochemical features. The patient is free-diseases by eight months.

** Key words:**Collagenous fibroma, desmoplastic fibroblastoma, neoplasm of connective and soft tissue.

## Introduction

Desmoplastic fibroblastoma (DF) is a rare benign neoplasm originated from fibroblastic and myofibroblastic cells described by Evans in 1995 (1). The denomination of collagenous fibroma, suggested by Nielsen in 1996 (2), depicts the histopathologic pattern of the lesion and has been used as a synonym. The etiology of DF is still uncertain, but recent cytogenetic analyses have detected a consistent 11q12 breakpoint which can lead to deregulated expression of FOSL1, a potential gene of pathogenetic importance (3). The occurrence in the oral cavity is very rare, and only nine cases have been described in the literature so far (4,5). The intraoral affected sites in the previously reported cases were the buccal mucosa (5,6), palate (4,7,8), gingiva (9), tongue (10) and alveolar bone (11). Clinically the lesion may present as a sessile or pedunculated, well circumscribed, firm, disk-shaped or lobulated mass which grows slowly and may reach 2 cm, but larger lesions have been described (4,7). Histopathologically, DF is composed of uniform stellate fibroblasts with large or oval nuclei and with myofibroblasts immersed in an abundant hypocellular dense collagen with variably myxoid stroma. Adjacent fat or skeletal muscle may be entrapped in some cases (1,7). Considering that oral cavity is a rare site to development of DF, this study describes a new case with unusual oral clinical presentation.

## Case Report

**Figure 1:**
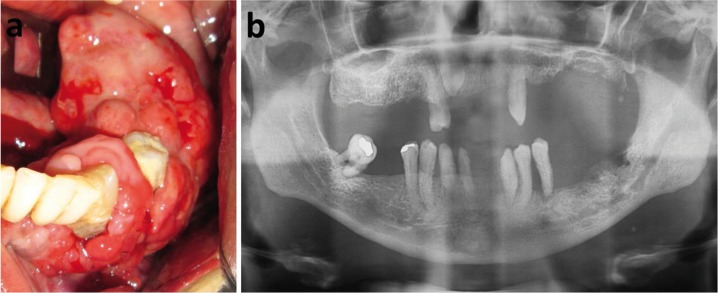
a) Clinical features of the desmoplastic fibroblastoma, extending from the retromolar region to the element 33. The lesion has lobulated surface and is covered by healthy mucosa with erythematous areas. Patient presented periodontal disease and poor oral hygiene. b) Panoramic radiograph shows irregular alveolar bone loss.

A 56-year-old man was referred to the Oral Medicine Clinic of Odilon Behrens Hospital to evaluate a mass in the left mandibular alveolar ridge, with slow and asymptomatic growth. The intraoral inspection revealed a sessile well-defined tumor with lobulated surface, covered by healthy mucosa with erythematous areas, extending from the retromolar region to the 33 tooth (Fig. 1). His medical, social and cultural histories were not contributory. The patient wore removable partial denture only in the upper jaw, presented advanced chronic periodontal disease in the lower arch, with mobility and dental calculus. The tumor was located in the edentulous region of the alveolar ridge without trauma related. Panoramic radiography showed irregular alveolar bone loss from area underlying the tumor to the 34 tooth (Fig. 1). Considering the clinical hypothesis of a benign mesenchymal neoplasia, an incisional biopsy was performed, and the specimen was sent to the Oral Pathology Department of the Universidade Federal Minas Gerais for histologic examination. With the previous diagnosis of benign fibrous lesion, a surgical excision was performed.

**Figure 2:**
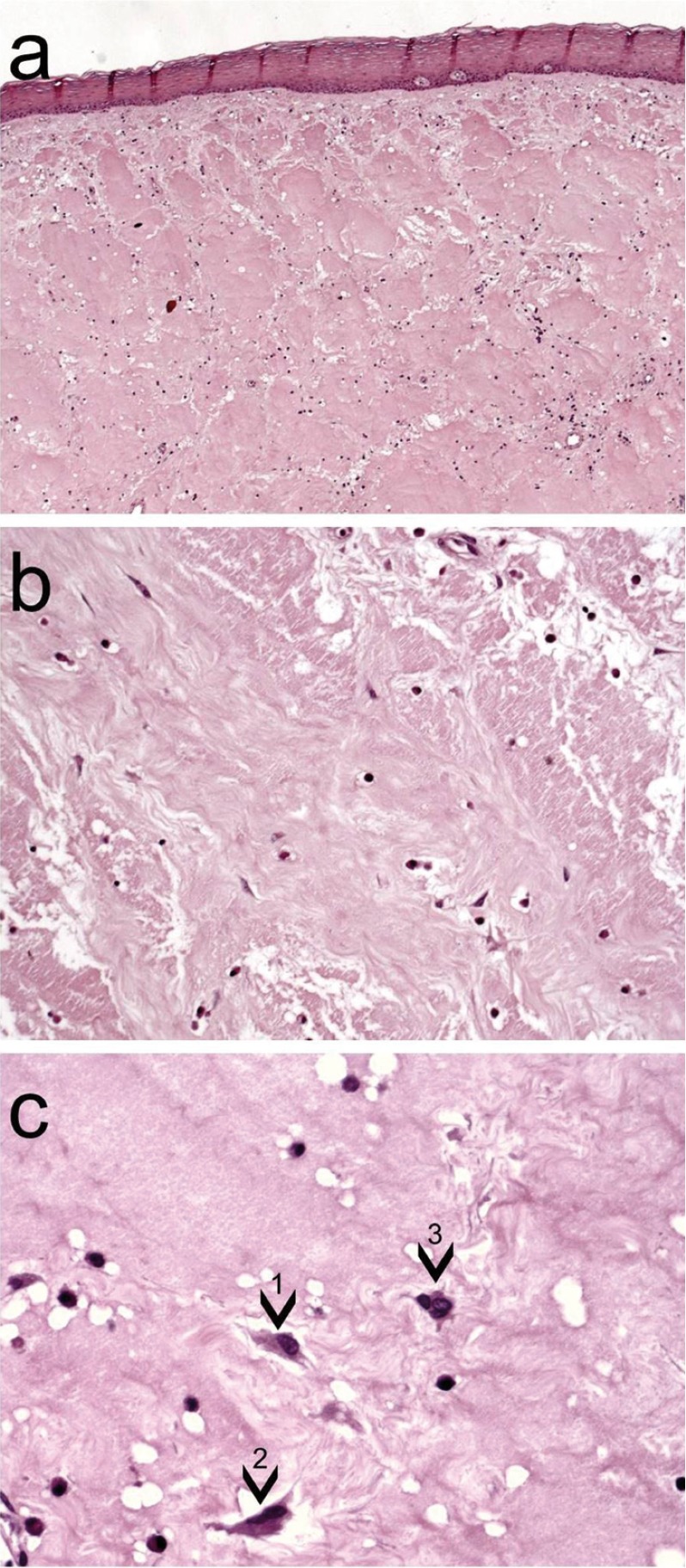
Histopathology of the desmoplastic fibroblastoma shows oral mucosa fragment with a non-encapsulated fibroblastic proliferation, (Haematoxylin and eosin, original magnification x50) a) characterized by spindle fibroblasts within a highly collagenous matrix (Hematoxylin and eosin, original magnification x100) b). Spindle to stellate shapes fibroblasts (1) presented large nuclei and binucleated (2) or (3) trinucleated fibroblasts were observed (Haematoxylin and eosin, original magnification x400) c).

**Figure 3:**
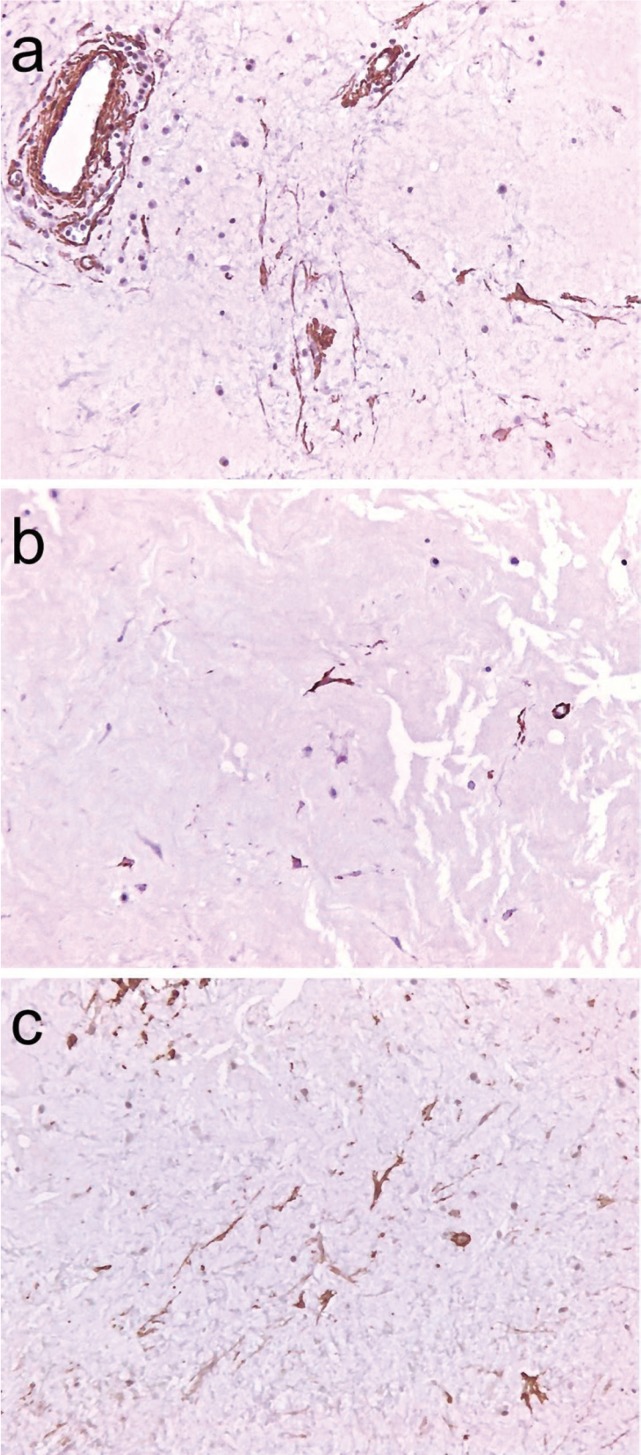
Immunohistochemistry demonstrated positive cells for α-smooth muscle actin a), HHF35 b), and factor XIIIa c) (Streptoavidin-biotin, x100 original magnification).

The specimen was fixed in 10% neutral formalin buffer and embedded in paraffin. Macroscopic examination revealed a tumor mass with gelatinous aspect in some areas after cross section of the surgical specimen. The microscopic exam showed a non-encapsulated fibroblastic proliferation, characterized by spindle or stellate fibroblasts within a highly collagenous matrix. Some fibroblasts presented bi or tri nucleation (Fig. 2). Immunohistochemical reactions were performed with the streptavidin method. Neoplastic cells showed immunopositivity for vimentin, HHF35, Factor XIIIa and a-smooth muscle actin (Fig. 3). The cells were immunonegative for S-100, AE1/AE3, CD34, and CD68. The final diagnosis was desmoplastic fibroblastoma.

The patient is on following and disease-free for eight months.

## Discussion

The case described in this report met the clinic-pathologic-immunohistochemistry features of DF and reveals a different intra oral site and clinical presentation from previously reported cases. A painless mass, with slow growth, covered by normal mucosa is the usual DF clinical aspect. In our case we observed a lobulated mass localized on alveolar ridge. DF arises despite no traumatic injury and no inciting event may be identified (7,8,10). The absence of a triggering event or a specific cause of a reactive fibrous proliferation corroborates with the hypothesis of a neoplastic process. Furthermore, the large size of the lesion exceeds the exte-sion that reactive proliferative lesions usually reach (1). Alveolar bone loss was not described in any case of oral DF. In the current case, panoramic radiograph shows irregular alveolar bone loss. Patient’s adult periodontitis may justify this bone involvement; however, it cannot affirm surely that this is the cause of bone loss not the tumor itself. Although the clinical aspect of DF is usually sessile (5-11), a pedunculated lesion was previously reported (4). DF shows predilection for females, with a mean age of 54 years (6) and the most frequent oral site is the palate (4,7,8). One case was described in an 8 year-old boy (5). The present case occurred in a 56-year-old man and was located on mandibular alveolar ridge. This oral site was not previously reported.

The DF´s histological differential diagnosis should be made with inflammatory fibrous hyperplasia (IFH), giant cell fibroma (GCF), traumatic fibroma (TF), benign mesenchymal neoplasms as myofibroma, neurofibroma and solitary fibrous tumor, also with less frequent soft tissue lesions as nodular fasciitis and fibromatosis (6,7). The presence of binucleated stellate fibroblasts and high hyalinized collagenous stroma contribute to the diagnosis of DF. On the other hand, the presence of inflammatory infiltrate and high cellularity suggests IFH, TF or GCF (6). The cellularity analysis is also useful to differentiate DF from fibromatosis. Although the lesions are histologically similar, fibromatosis is more cellular than DF, showing a diffuse cellularity (12). Binucleated or stellate shaped fibroblasts are rarely found in IFH, TF and GCF (13). The entrapment of adjacent fat and skeletal muscle is a common histologic feature described in previous reports of DF, however this phenomenon was not observed in this case (7,13). The region of alveolar ridge is free of the fat or skeletal muscle and this may be explained by the absence of this tissue in the current case. Extensive areas of hyalinized collagenous and sclerosis are common to DF and solitary fibrous tumor, however the second shows hypercellular areas that are not found in DF (14).

Immunohistochemical panels provide evidence for differentiation of these lesions and are important to exclude lesions of others origin. However, it is not mandatory to DF diagnosis. GCF is negative for α-smooth muscle actin and rarely positive for factor XIIIa (15), in contrast DF is positive for both proteins (7). Nodular fasciitis is positive for α-smooth muscle actin, muscle-specific actin (HHF35) and desmin (16), different from DF which is negative for desmin. The immunohistochemistry profile of DF reveals a predominantly fibroblastic origin with focal myofibroblastic differentiation. DF cells are usually negative to S-100 protein, CD34, desmin, cytokeratins, epithelial membrane antigen and CD68, discarding the possibility of neural, vascular, or epithelial origin (6). Tumor cells may show diffuse positivity for vimentin and focal immunopositivity for HHF35, Factor XIIIa (6,7) and α-smooth muscle actin (7,8,14) as observed in this case.

Cytogenetic analyses suggest that rearrangement of chromosome band 11q12 is recurrent and associated with DF, however no target gene has been described as diagnostic tool. Most recent evidence indicates that the FOSL1 gene is overexpressed in DF due to this chromosomal rearrangement (3). Although translocations involving chromosome band 11q12 are associated with DF, this analysis was not performed in tumors from oral cavity.

The current case study demonstrated a clinic-pathologic-immunohistochemistry of DF localized oral cavity, a rare fact. It is important to discuss features new of oral DF providing more know about this disease.
